# Protein tyrosine phosphatase PTPN22 is dispensable for dendritic cell antigen processing and promotion of T-cell activation by dendritic cells

**DOI:** 10.1371/journal.pone.0186625

**Published:** 2017-10-17

**Authors:** Fiona Clarke, Christine K. Jordan, Enrique Gutiérrez-Martinez, Jack A. Bibby, Cristina Sanchez-Blanco, Georgina H. Cornish, Xuezhi Dai, David J. Rawlings, Rose Zamoyska, Pierre Guermonprez, Andrew P. Cope, Harriet A. Purvis

**Affiliations:** 1 Academic Department of Rheumatology, Centre for Inflammation Biology and Cancer Immunology, Faculty of Life Sciences and Medicine, King’s College London, London, United Kingdom; 2 Department of Immunobiology, Centre for Inflammation Biology and Cancer Immunology, Faculty of Life Sciences and Medicine, King’s College London, London, United Kingdom; 3 Seattle Children’s Research Institute and Departments of Pediatrics and Immunology, University of Washington School of Medicine, Seattle, Washington, United States of America; 4 Institute of Immunology and Infection Research, Centre for Immunity, Infection and Evolution, University of Edinburgh, Edinburgh, United Kingdom; Baylor College of Medicine, UNITED STATES

## Abstract

The PTPN22^R620W^ single nucleotide polymorphism increases the risk of developing multiple autoimmune diseases including type 1 diabetes, rheumatoid arthritis and lupus. PTPN22 is highly expressed in antigen presenting cells (APCs) where the expression of the murine disease associated variant orthologue (Ptpn22^R619W^) is reported to dysregulate pattern recognition receptor signalling in dendritic cells (DCs) and promote T-cell proliferation. Because T-cell activation is dependent on DC antigen uptake, degradation and presentation, we analysed the efficiency of these functions in splenic and GM-CSF bone marrow derived DC from wild type (WT), *Ptpn22*^*-/-*^ or *Ptpn22*^*R619W*^ mutant mice. Results indicated no differential ability of DCs to uptake antigen via macropinocytosis or receptor-mediated endocytosis. Antigen degradation and presentation was also equal as was WT T-cell conjugate formation and subsequent T-cell proliferation. Despite the likely presence of multiple phosphatase-regulated pathways in the antigen uptake, processing and presentation pathways that we investigated, we observed that Ptpn22 and the R619W autoimmune associated variant were dispensable. These important findings indicate that under non-inflammatory conditions there is no requirement for Ptpn22 in DC dependent antigen uptake and T-cell activation. Our findings reveal that perturbations in antigen uptake and processing, a fundamental pathway determining adaptive immune responses, are unlikely to provide a mechanism for the risk associated with the Ptpn22 autoimmune associated polymorphism.

## Introduction

The C1858T polymorphism within the protein tyrosine phosphatase *PTPN22* (encoding PTPN22^R620W^) is a major risk factor for the development of multiple autoimmune diseases, including rheumatoid arthritis (RA), type I diabetes, lupus and juvenile idiopathic arthritis (JIA) [1]. PTPN22 (Ptpn22 in the mouse) negatively regulates Src and Syk family kinase (SFK) activity downstream of the T-cell antigen receptor (TCR) [2]. As a result, T-cells from *Ptpn22*^-/-^ mice exhibit enhanced TCR signalling resulting in homeostatic expansion of CD4^+^ effector T-cells [3]. In addition to TCR signalling, PTPN22 regulates many pathways in different cell types including the B-cell receptor [4], the αLβ2 integrin LFA-1 [5] and Toll-Like Receptor (TLR) signalling pathways [6–9]. Furthermore, Ptpn22 also functions to alter SFK independent signalling events by modulating TRAF ubiquitination [8]. It has become widely accepted that the autoimmune associated *PTPN22*^R620W^ variant displays reduced binding to the tyrosine kinase Csk, due to a missense mutation in the P1 domain [2,10], but precisely how the PTPN22-R620W variant affects the functions of immune cell subsets is more complex. Both gain- and loss-of-phosphatase function effects have been reported, depending on the cellular context and signalling pathway under investigation [4,5,9–11].

Antigen presenting cells (APCs) are critical for pathogen sensing and antigen uptake to effectively clear infection. Ptpn22 is highly expressed in myeloid cells and a functional role for Ptpn22 in the regulation of multiple pattern recognition receptors has been established [6,8,9]. Antigen uptake occurs via non-specific pinocytosis or via receptor-mediated phagocytosis, and is critical for APC mediated T-cell activation. Despite this, investigations assessing the capability of Ptpn22 to regulate antigen uptake and presentation are limited. A recent study reported that Ptpn22^R619W^ (the murine orthologue of the human autoimmune associated variant R620W) expression in macrophages under non-inflammatory conditions increased wild type (WT) OT-II T-cell activation and proliferation. It was suggested that this was caused by an enhanced phagocytic capability of Ptpn22^R619W^ macrophages [12]. A further study documented that Ptpn22^R619W^ expressing BMDC matured with LPS also induce enhanced WT OT-II T-cell proliferation compared to WT controls, attributed to enhanced co-stimulation rather than phagocytosis [9]. Src and Syk family kinases regulate multiple processes that determine antigen uptake, processing and presentation, and ultimately, the capability of APCs to activate T-cell responses [13]. For example, phosphorylation of FcγR and dectin-1 associated ITAM motifs by SFKs regulates receptor-mediated phagocytosis [14–16], whilst SFKs also mediate tyrosine phosphorylation of cortactin leading to cytoskeletal rearrangement which facilitates engulfment of antigen [14]. Enhanced antigen phagocytosis and processing may, in addition, lead to enhanced T-cell activation, thus further contributing to the expansion of autoreactive T-cells *in vivo* [4,11]. We therefore sought to systematically assess the role of Ptpn22 in antigen phagocytosis and processing, and its impact on T-cell activation.

## Methods

### Ethics statement

All animal work has been conducted according to The Animals (Scientific Procedures) Act 1986 under Home Office License 70/8792. The King’s College London Animal Welfare and Ethical Review Body (AWERB) approved the research. Animals were sacrificed by a rising CO_2_ concentration and death was confirmed by cervical dislocation.

### Mice

Wild type (WT) C57BL/6, *Ptpn22*^-/-^, *Ptpn22*^R619W^ mice, OT-II, and OT-II x Ly5.2 (CD45.1) were housed under specific pathogen free (SPF) conditions and used in experiments according to UK Home Office approved protocols. *Ptpn22*^-/-^ mice and *Ptpn22*^R619W^ mutant mice were backcrossed for more than 10 generations to the C57BL/6 strain. Their generation, genotype and phenotype have been previously described in detail [4,17] Age and gender-matched mice were used in all experiments.

### Bone marrow derived dendritic cell (BMDC) culture

BMDC were produced using a protocol adapted from Inaba *et al* [18]. Bone marrow was flushed from femurs and tibias of WT, *Ptpn22*^-/-^ or *Ptpn22*^*R619W*^ mice with RPMI-1640 supplemented with L-glutamine (Corning) containing 1% FBS and penicillin/streptomycin (100 μg/ml). Bone marrow cells were incubated for 30 minutes at 37°C in 5% CO_2_ on Petri dishes to remove adherent macrophages. Non-adherent progenitor cells were seeded at 1.5 x 10^6^ cells/ml in 24-well tissue culture plates in RPMI-1640 with L-glutamine supplemented with 10% heat-inactivated FBS, β-mercaptoethanol (50 μM), penicillin/streptomycin (100 μg/ml) and 1% murine GM-CSF; GM-CSF was produced from the B78H1/GMCSF.1 cell line. BMDC were cultured for 6 days at 37°C and 5% CO_2_ and medium replaced on days 3 and 4. BMDCs were used in functional assays on days 6 and 7.

### BMDC phenotype

BMDCs were harvested and stained using anti-mouse CD11c-PE/Cy7 (clone N418; Biolegend), anti-mouse CD11b-APC/Cy7 (clone M1/70; Biolegend), MHC class II I-A^b^-FITC (clone AF6-120.1; Biolegend), CD206-PE (clone C068C2; Biolegend) and Zombie UV Fixable Viability (Biolegend) in PBS containing anti-mouse CD16/CD32 (clone 93; Biolegend). Cells were washed with FACS buffer (5% FBS, 1 mM EDTA, 0.01% NaN_3_ in PBS) and fixed using 1% paraformaldehyde (PFA, Electron Microscopy Services) in PBS.

### Pinocytosis assay

BMDC were stimulated overnight in the presence or absence of LPS (100 ng/ml, Invivogen). BMDC (2 x 10^5^ cells/FACS tube) were incubated with Lucifer yellow (LY, Invitrogen, 1 mg/ml) at 37°C for the indicated time points. Samples were returned to ice and washed twice with cold PBS to prevent further uptake. Cells were stained with anti-mouse CD11c-PE/Cy7 and Zombie UV Fixable Viability dye for 30 minutes on ice. Cells were washed with FACS buffer and fixed using 1% PFA in PBS.

### Receptor-mediated endocytosis assay

BMDC (2 x 10^5^) were added to FACS tubes and incubated with ovalbumin-AF488 (Invitrogen, 10 μg/ml) or Zombie UV Fixable Viability dye labelled heat killed *Listeria monocytogenes* (HKLM, Invivogen, 1 DC:30 HKLM) on ice for 45 minutes. Cells were washed with cold FACS buffer and incubated at 37°C for various time points. Samples were returned to ice and washed with cold PBS to prevent further uptake. Cells were stained with anti-mouse CD11c-PE/Cy7 and Fixable Viability Dye eFluor 506 (eBioscience) for 30 minutes on ice. Cells were washed with FACS buffer and fixed using 1% PFA in PBS.

### Antigen uptake by splenic DCs

Spleens from WT, *Ptpn22*^-/-^ or *Ptpn22*^*R619W*^ mice were digested in 1 ml of RPMI 1640, containing DNase and Liberase (both at 0.1mg/ml, Roche) on 24-well plates for 30 minutes at 37°C in 5% CO_2_; 10 mM EDTA was added for the final 5 minutes. Splenocyte cell suspensions were resuspended in 2 ml of red blood cell lysis buffer (Biolegend), washed in PBS and pelleted (1600 rpm, 5 minutes). 5 x 10^6^ splenocytes were added per FACS tube. Splenocytes were cooled and incubated with ovalbumin-AF647 (Invitrogen, 50 μg/ml) for 45 minutes on ice. Cells were washed and transferred to 37°C for 45 minutes to allow for uptake. Cells were stained to identify splenic DCs and were identified as singlet, live, lineage^-^ (excluding CD3, CD19, Ter119, NK1.1, GR1, B220^+^ splenocytes), CD11c-PE/Cy7^+^, MHC class II I-A^b^-FITC^+^.

### Antigen processing assay

3 μm polystyrene beads (Polysciences) were coated overnight at 4°C with ovalbumin-AF594 (Invitrogen, 0.5 mg/ml). 1 x 10^5^ BMDC and 2 x 10^6^ beads were added to duplicate wells of a 96-well round bottom plate (Nunc, 1 DC:20 beads) and pelleted (2000 rpm, 5 minutes). BMDC were incubated at 37°C, 5% CO_2_ for 0–5 hour time points. To exclude non-internalised beads, cells were stained with rabbit anti-ovalbumin (Sigma, 50 μg/ml) for 20 minutes on ice, then washed and stained with F(ab’)2 goat anti-rabbit IgG-AF647 (Invitrogen, 4 μg/ml) for 20 minutes on ice. Cells were washed with FACS buffer and lysed for 10 minutes on ice (0.5% NP-40, 50 mM Tris, 150 mM NaCl). Beads were washed twice with FACS buffer and transferred to FACS tubes.

### CD4^+^ T-cell isolation

MACS negative selection kits (Miltenyi Biotech) were used to isolate CD4^+^ T-cells from the lymph nodes (LN) and spleens of 8–16 week old WT OT-II or WT OT-II CD45.1 mice. T-cells (2 x 10^7^ cells/ml) were labelled with 2 μM CellTrace Violet (CTV, Invitrogen) for 20 minutes at 37°C.

### DC:T-cell conjugate assay

BMDC were incubated overnight with LPS (100 ng/ml, Invivogen) and OVA_323-339_ peptide (10 μM, Invivogen). Cells were harvested, washed and resuspended at 1 x 10^7^/ml in PBS prior to staining with 1 μM CellTrace Far Red (CTFR, Invitrogen) for 20 minutes at 37°C, followed by quenching in culture media for 20 minutes at 37°C. 1 x 10^5^ CTFR labelled BMDC and 2 x 10^5^ CTV labelled WT OT-II T-cells (in a total volume of 50μl) were added to each 1.5 ml tube, centrifuged for 2 minutes at 500 rpm and incubated at 37°C for the indicated times. Cells were fixed with 3% PFA in PBS for 15 minutes at room temperature, transferred to FACS tubes and acquired using a medium flow rate on a FACS Canto (BD). Conjugates were identified as CTV^+^ CTFR^+^ cells.

### Antigen presentation assay

2 x 10^5^ BMDC were incubated on 96-well U bottom plates with 0.3 mg/ml GFP-Eα (a kind gift from Erwan Boëdec, Paris 7 University) for 18 hours at 37°C. BMDC were harvested, washed and stained with anti-CD11c-PE/Cy7, anti-Eα_52-68_-biotin (eBioscience) and Fixable Viability Dye eFluor 506 for 30 minutes on ice. Cells were washed with FACS buffer and stained with PBS containing streptavidin-APC (Biolegend) at room temperature for 10 minutes. Cells were washed and fixed in 1% PFA in PBS prior to flow cytometry. BMDC were identified as live, CD11c^+^ singlets.

### *In vitro* co-cultures

WT and *Ptpn22*^*-/-*^ BMDC were pulsed overnight with ovalbumin (Invivogen) or OVA_323-339_ (Invivogen) at 0.01–1 μM. BMDC were harvested, washed and resuspended in RPMI-1640 with L-glutamine supplemented with 10% heat-inactivated FBS, β-mercaptoethanol (50 μM), penicillin/streptomycin (100 μg/ml). CD4^+^ T-cells were isolated and CTV labelled as described above. BMDC were co-cultured with CTV labelled CD4^+^ T-cells at 1:2 BMDC:T-cell ratio (1 x 10^5^ BMDC:2 x 10^5^ T-cells) in 96-well U bottom plates for 1–6 days.

### T-cell adoptive transfer

1 x 10^6^ CTV labelled CD4^+^ TCR Vα2^+^ Vβ5^+^ WT OT-II T-cells were injected intravenously into WT or *Ptpn22*^*-/-*^ mice. The following day mice were immunised with ovalbumin (10 μg/mouse) or PBS via intraperitoneal injection. After 72 hours spleens were harvested and single cell suspensions were stained using antibodies against: TCR Vα2-PE (clone MR9-4; Biolegend), TCR Vβ5-APC (clone B20.1; Biolegend), CD4-APC/Cy7 (clone GK1.5; Biolegend), CD45.1-PE/Cy7 (clone A20; Biolegend), CD45.2-BV785 (clone 104; Biolegend). CTV dilution was determined within the live, singlet, CD4^+^, Vα5^+^Vβ2^+^, CD45.1^+^, CD45.2^-^ population.

### Cytokine immunoassays and cell phenotyping

Cell-free co-culture supernatants were harvested at the indicated times. IL-2 and IFNγ were determined by immunoassay. Antibody pairs were purchased from Biolegend. Cytokine levels were determined using streptavidin-europium and enhancement solution (both from Perkin Elmer) and detected on a Victor 1420 multilabel counter (Perkin Elmer). Day 1 or 6 BMDC and OT-II T-cell co-cultures were harvested and stained with anti-CD3ε-FITC (clone 145.2C11; Biolegend), anti-CD4-PerCP (clone RM4-5; Biolegend), anti-CD69 (clone H1.2F3; Biolegend), anti-CD25 (PC61; Biolegend) and Fixable Viability Dye eFluor 506. Cells were fixed in 1% PFA in PBS. Proliferating and activated T-cell populations were determined gating on live, singlet, CD3^+^, CD4^+^ cells and by CTV dilution.

### Flow cytometry

Gates were determined by fluorescence minus one (FMO) controls. Cells were acquired using Becton Dickinson Fortessa or FACSCanto II flow cytometers and data analysed using FlowJo Version 8.7.

### BMDC genotyping

Day 7 BMDCs were lysed in 50mM Tris (pH 8), 25mM EDTA, 100mM NaCl, 1% SDS and 0.4mg/ml Proteinase K (Roche) 1 hour at 56°C. DNA was extracted and a PCR was carried out using the following conditions: 94°C for 1min, followed by 29 cycles of 94°C for 15 seconds, 55°C for 15 seconds, 72°C for 12 seconds, all followed by a 10 minute extension step at 72°C. The following primers were used: 5'-AGCCAAGTTTCTTTGTTGAGAA-3' and 5'-CAGACACAACAAAGCCCAGA-3' (Sigma). PCR products were run on a 1% agarose gel and WT and *Ptpn22*^-/-^ bands were identified (498bp and 221bp respectively).

### Real-time PCR

Total RNA was extracted from BMDCs using TRIzol reagent, and cDNA was reverse transcribed using first strand cDNA synthesis using random hexamers. Gene expression was measured by TaqMan quantitative real-time PCR using FAM labelled Ptpn22 (Mm00501246_m1; Applied Biosystems) and VIC labelled 18S probe. Acquisition was conducted using the ABI 7900HT fast real-time PCR system (Applied Biosystems). Relative abundance of gene expression was calculated using 18S as an endogenous control, and the formula: relative abundance = 2^(-dCt)^.

### Statistical analysis

GraphPad Prism software was used for statistical analysis by unpaired T-test or two-way ANOVA with Sidak’s post-test (paired or unpaired; two-tails).

## Results

### Ptpn22 does not impact antigen uptake via macropinocytosis

**Fig 1:**
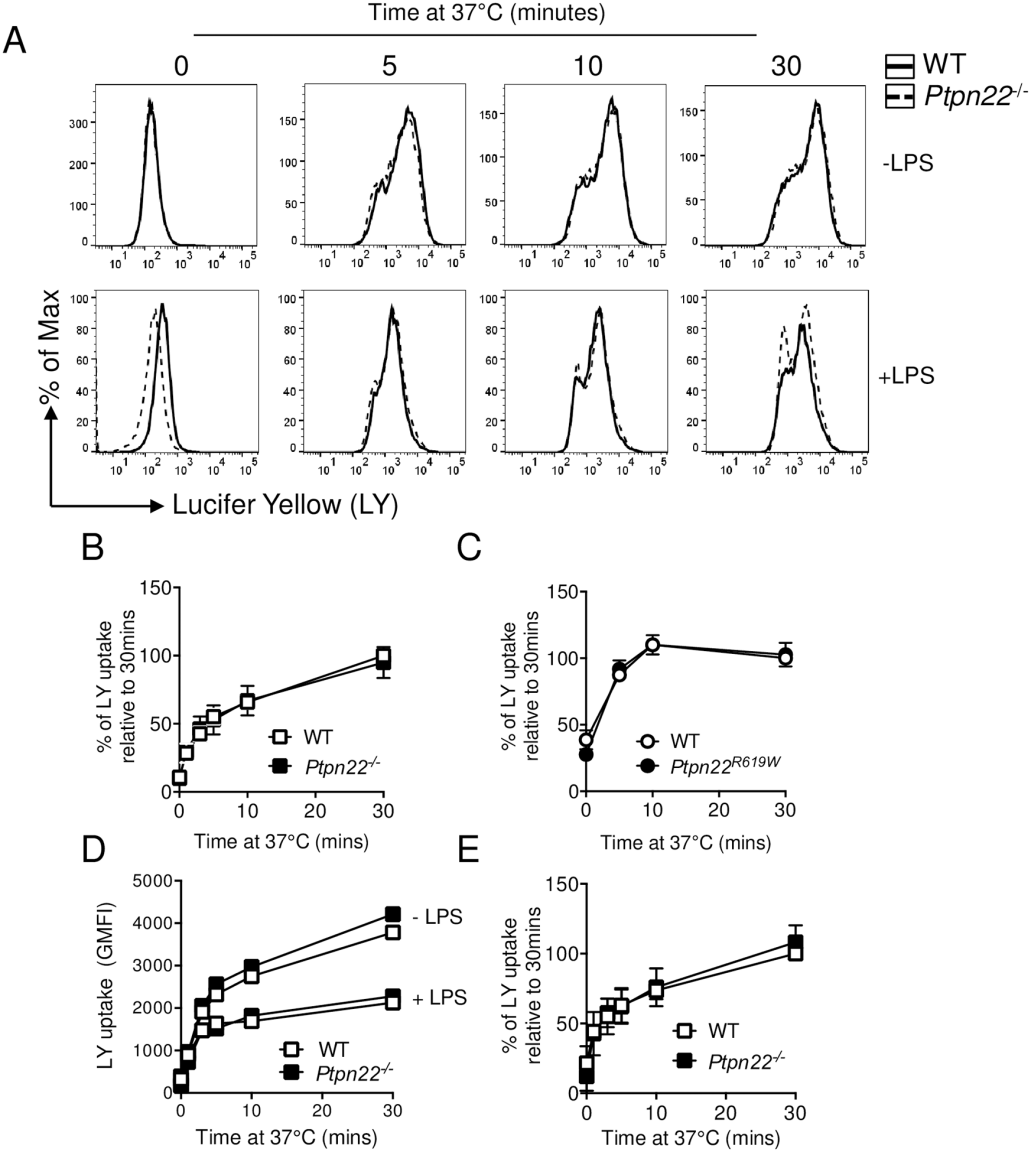
Macropinocytosis does not require Ptpn22. **(A-E)** Wild type (WT), *Ptpn22*^-/-^ and *Ptpn22*^*R619W*^ derived bone marrow derived dendritic cells (BMDC) were harvested and stimulated at 37°C for 0–30 minutes with Lucifer Yellow (LY). LY expression was determined by flow cytometry by gating on live, singlet, CD11c^+^ cells. **(A)** Representative flow cytometry plots showing (LY) uptake by WT (black solid line) and *Ptpn22*^*-/-*^ (black dashed line), immature (top row) and LPS matured BMDC (bottom row). **(B)** LY uptake by WT (white square) and *Ptpn22*^*-/-*^ (black square) immature BMDC. Data shown are Geometric Mean Fluorescent Intensity (GMFI) relative to maximal WT uptake at 30 minutes; N = 4 independent experiments; line represents mean ± s.d. **(C)** LY uptake by WT (white circle) and *Ptpn22*^*R619W*^ (black circle) immature BMDC. Data shown are GMFI relative to maximal WT uptake at 30 minutes; N = 3 independent experiments; line represents mean ± s.d. **(D)** LY uptake by immature (-LPS) or LPS matured (+LPS) WT (white square) and *Ptpn22*^*-/-*^ (black square) BMDC, showing representative LY GMFI of 3 independent experiments. **(E)** LY uptake relative to maximal WT uptake at 30 minutes by LPS matured WT (white square) and *Ptpn22*^*-/-*^ (black square) BMDC. Data shown are GMFI mean ± s.d; N = 3 independent experiments.

Macropinocytosis occurs constitutively in APCs allowing for non-specific uptake of soluble molecules, nutrients and antigens [14,19]. Pinocytosis requires actin-dependent membrane ruffling, ultimately leading to MHC class I and MHC class II dependent antigen presentation to T-cells [19–21]. First, we assessed if Ptpn22 variants could regulate antigenic uptake via pinocytosis. To address this, we generated BMDC in the presence of GM-CSF from WT, *Ptpn22*^*-/-*^ and *Ptpn22*^*R619W*^ mice; Ptpn22 expression was verified by genotyping and PCR (Panels A and B in S1 Fig). No genotype specific differences were observed with respect to the proportion or number of CD11c^+^ DC, (or macrophage like CD11c^+^, CD11b^Hi^, I-A^bInt^ cells) generated within these cultures (Panels A and B in S2 Fig) [14]. Macropinocytosis was assessed by the capability of WT and *Ptpn22*^*-/-*^ BMDC to take up Lucifer yellow (LY). WT and *Ptpn22*^*-/-*^ BMDC were incubated in the presence of LY at 37°C for time points up to 30 minutes. As expected, the LY geometric mean fluorescent intensity (GMFI) signal increased over time (Fig 1A), and internalisation of LY by WT, Ptpn22^R619W^, and *Ptpn22*^*-/-*^ BMDC was comparable (Fig 1A–1C). Following TLR mediated maturation, macropinocytosis is decreased [14,21,22]. Accordingly, we assessed if the absence of Ptpn22 altered the capability of BMDC to downregulate LY uptake following LPS mediated maturation. We observed that, as previously reported [21], BMDC pinocytosis was significantly reduced following LPS treatment (Fig 1D), however WT and *Ptpn22*^*-/-*^ BMDC had an equal reduction in LY uptake (Fig 1D and 1E). We therefore conclude that altered Ptpn22 function does not impact macropinocytosis.

### Ptpn22 does not influence mannose receptor-mediated antigen uptake

**Fig 2:**
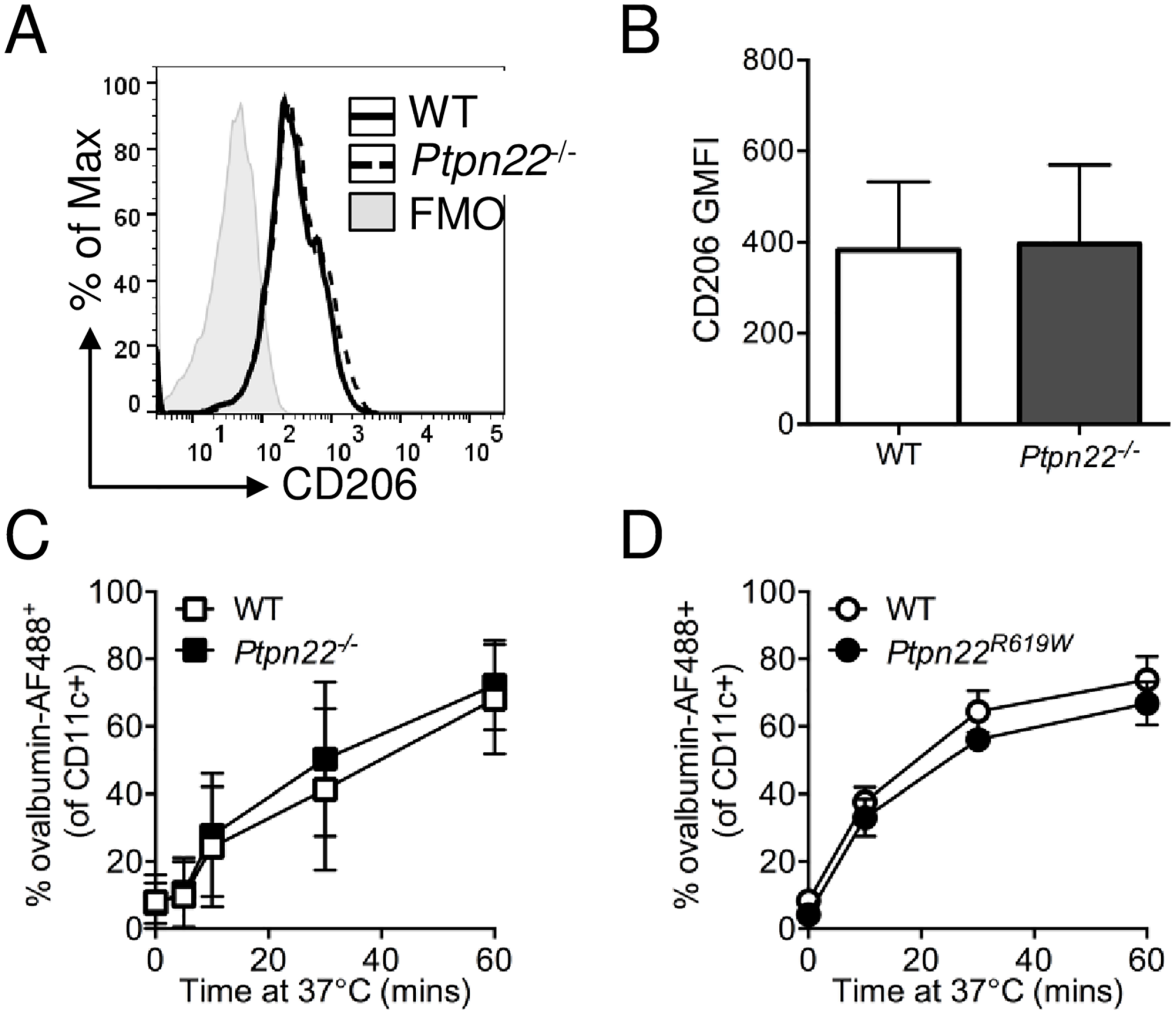
Ptpn22 variants do not alter BMDC receptor mediated phagocytosis. **(A-B)** Wild type (WT) and *Ptpn22*^-/-^ derived bone marrow derived dendritic cells (BMDC) were harvested and cell surface CD206 was determined by flow cytometry. **(A)** Representative CD206 expression profiles on WT (black solid line) and *Ptpn22*^*-/-*^ (black dashed line) BMDC. **(B)** CD206 Geometric Mean Fluorescent Intensity (GMFI). N = 11 independent experiments; bars represent mean ± s.e.m. **(C)** WT and *Ptpn22*^*-/-*^ BMDC were incubated with ovalbumin-AF488 for 0–60 minutes at 37°C prior to CD11c staining. N = 4 independent experiments; line represents the mean ± s.d. **(D)** WT and *Ptpn22*^*R619W*^ BMDC were incubated with ovalbumin-AF488 for 0–60 minutes at 37°C prior to CD11c staining. N = 3 independent experiments; line represents the mean ± s.d.

Phagocytic cell surface receptors expressed on APCs mediate uptake of specific types of antigen through recognition of specific pattern associated molecular patterns (PAMPs) [23]. Receptor-mediated endocytosis is dependent on a number of processes including receptor clustering, modulation of specific signalling pathways by ubiquitination and kinase-induced phosphorylation, in addition to actin and cytoskeletal rearrangement [23,24]. We assessed the involvement of Ptpn22 in mannose receptor (CD206) dependent phagocytosis of ovalbumin [24], since ovalbumin provides an ideal model with which to assess the impact of altered phagocytosis on antigen specific OT-II T-cell activation. We confirmed that there was no difference in cell surface expression of CD206 on immature WT versus *Ptpn22*^*-/-*^ BMDC (Fig 2A and 2B) and WT versus *Ptpn22*^*R619W*^ BMDC (Panel A in S3 Fig). We next assessed if binding and uptake of ovalbumin conjugated to AF488 (ovalbumin-AF488) was regulated by Ptpn22. Wild type and *Ptpn22*^*-/-*^ BMDC were incubated with ovalbumin-AF488 at 37°C for up to 60 minutes and the presence of AF488 within CD11c^+^ cells determined. We observed over time that the proportion of AF488^+^ BMDC increased, but no differences were observed between the WT and *Ptpn22* deficient BMDC (Fig 2C), or between WT and *Ptpn22*^*R619W*^ BMDC (Fig 2D) at any time point. A lack of specific signalling motifs within the CD206 cytoplasmic domain suggests that CD206 dependent antigen uptake may not be directly dependent on SFKs [25], despite the requirement of SFKs in initiating initial cortical actin protrusions involved in early phagocytic events [26]. Therefore we assessed if Ptpn22 regulates the uptake of antigens via other pattern recognition receptors, such as TLRs. *Listeria monocytogenes* is phagocytosed via TLR2, and *Candida albicans* via dectin-1, both in a SFK dependent manner [26]. We observed no significant difference in the capability of WT or *Ptpn22*^-/-^ BMDCs to uptake heat killed *L*. *monocytogenes* (Panel B in S3 Fig) or heat killed *C*. *albicans* (HKCA) (Panel C in S3 Fig). Together these data indicate that despite the presence of phosphatase dependent pathways, Ptpn22 does not impact antigen uptake via multiple phagocytic receptor pathways.

### Splenic dendritic cell uptake of ovalbumin occurs independently of Ptpn22

**Fig 3:**
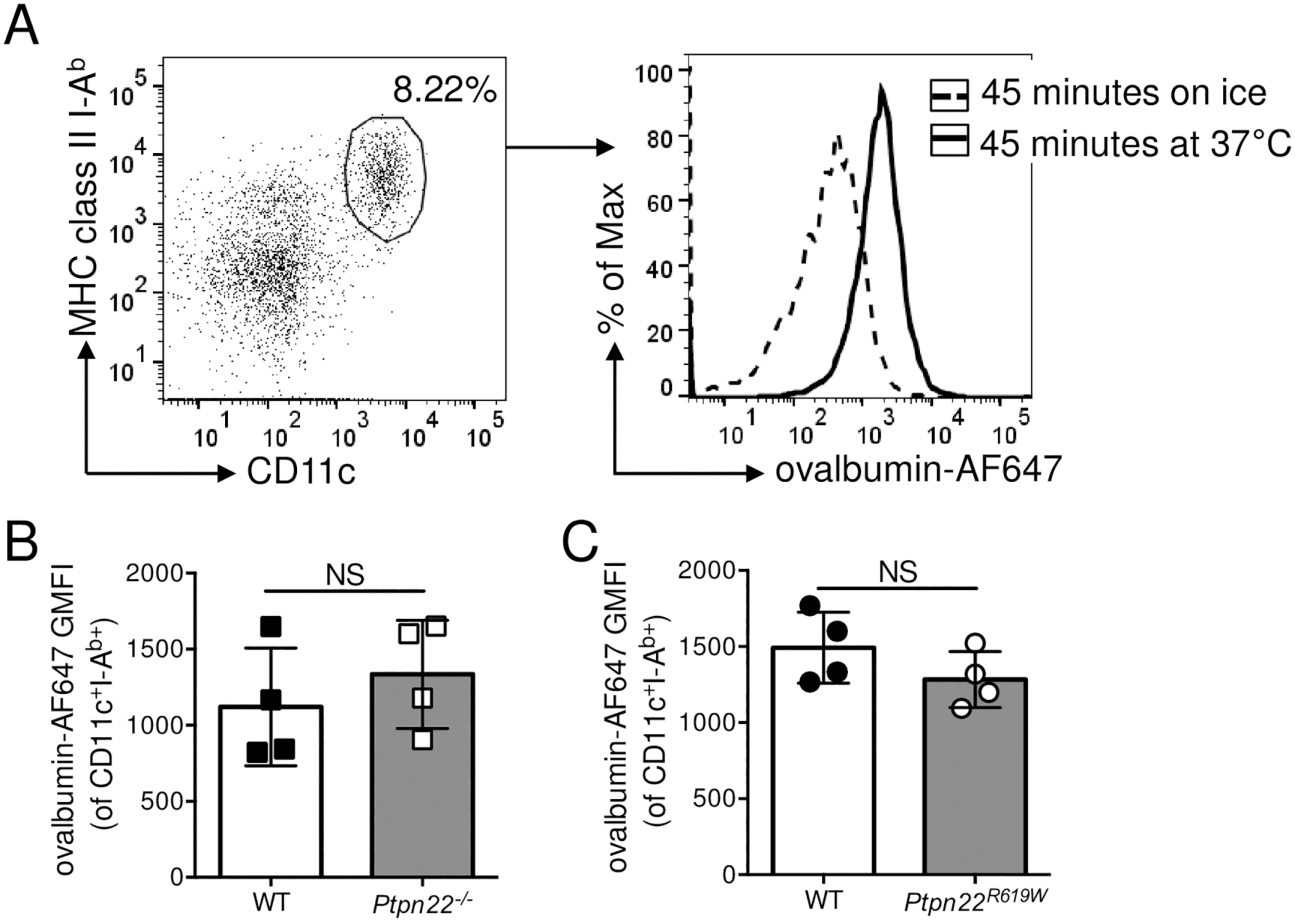
Regulation of splenic DC receptor mediated phagocytosis of ovalbumin occurs independently of Ptpn22. **(A-B)** Wild type (WT) and *Ptpn22*^-/-^ splenocytes were incubated for 45 minutes with ovalbumin-AF647 on ice or at 37°C and stained for lineage^-^, CD11c^+^, I-A^b+^ DC. **(A)** Representative flow cytometry staining of CD11c^+^ I-A^b+^ splenic DCs (left hand panel, gated on live, singlet, lineage^-^) and ovalbumin-AF647 fluorescence in splenic DCs (right hand panel) incubated with ovalbumin-AF647 for 45 minutes on ice (black dashed line) or at 37°C (black solid line). **(B)** ovalbumin-AF647 Geometric Mean Fluorescent Intensity (GMFI) of WT and *Ptpn22*^*-/-*^ CD11c^+^ I-A^b+^ splenic DC incubated at 37°C for 45 minutes. Squares represent individual mice and bars represent the mean ± s.d. **(C)** Wild type (WT) and *Ptpn22*^*R619W*^ splenocytes were incubated for 45 minutes with ovalbumin-AF647 at 37°C. Circles represent individual mice and bars represent the mean ± s.d. NS = not significant by unpaired T-test.

*In vitro* generated BMDC are a widely used tool to assess APC function, but differ from *in vivo* DC subsets. To address if *in vivo* ovalbumin antigen uptake by DCs was regulated by *Ptpn22*, we isolated the spleens of WT, *Ptpn22*^-/-^ and *Ptpn22*^*R619W*^ mice, and incubated splenocytes with ovalbumin-AF647 for 45 minutes either on ice or at 37°C. Following 45 minutes at 37°C there was an increase in the ovalbumin-AF647 GMFI of CD11c^+^ MHC class II^+^ splenic DCs (Fig 3A). However, when we assessed binding and uptake of ovalbumin-AF647 by WT, *Ptpn22*^*-/-*^, or *Ptpn22*^*R619W*^ CD11c^+^ MHC class II^+^ splenic DCs we observed no differences (Fig 3B and 3C), indicating that, as observed with *in vitro* generated BMDC, the capability of splenic DC to take up ovalbumin is not dependent on Ptpn22.

### Ptpn22 is dispensable for antigen processing and presentation

**Fig 4:**
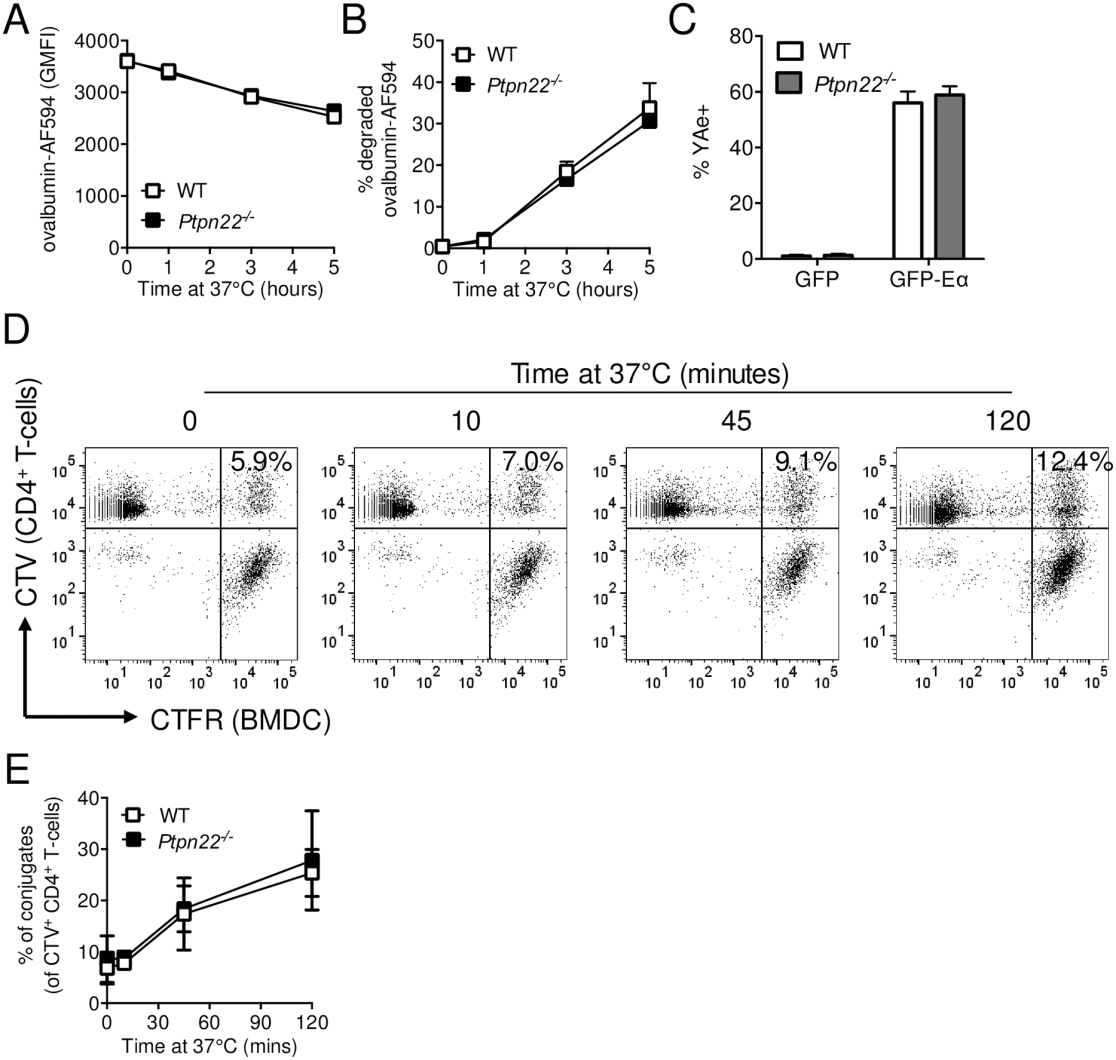
Ptpn22 is dispensable for antigen degradation and presentation. **(A-B)** Wild type (WT) and *Ptpn22*^*-/-*^ BMDC were incubated with ovalbumin-AF594 coated beads for 0–5 hours at 37°C. Non-internalised beads were excluded by staining with rabbit anti-ovalbumin followed by F(ab’)_2_ anti-rabbit-AF647. Cells were lysed and the fluorescent intensity of AF594^+^ AF647^-^ beads determined by flow cytometry. **(A)** Ovalbumin-AF594 Geometric Mean Fluorescent Intensity (GMFI). N = 2 independent experiments ± s.d. **(B)** Proportion of internalised beads from WT and *Ptpn22*^*-/-*^ BMDC that have reduced ovalbumin-AF594 fluorescence. N = 2 independent experiments ± s.d. **(C)** WT and *Ptpn22*^*-/-*^ BMDC were incubated with GFP or GFP-Eα for 18 hours followed by staining for Eα_52–68_ in I-A^b^ (YAe). The percentage of YAe^+^ live, singlet, CD11c^+^ BMDC was determined by flow cytometry. N = 3 independent experiments; bars represent mean ± s.d. **(D-E)** CellTrace Violet (CTV) labelled WT CD4^+^ OT.II T-cells were incubated with CellTrace Far Red (CFTR) stained OVA_323-339_ peptide pulsed LPS matured WT or *Ptpn22*^*-/-*^ BMDC for 0–120 minutes at 37°C, and the proportion of CTV^+^ CTFR^+^ conjugates within the CTV^+^ T-cell population determined by flow cytometry. **(D)** Representative flow cytometry plots showing conjugates (top, right hand quadrant, CTV^+^ CTFR^+^). **(E)** Proportion of DC:T-cell conjugates within CTV^+^ gate. N = 4 independent experiments; line represents mean ± s.d. Data **(C)** determined non-significant by unpaired T-test. Differences between genotypes in **(E)** were deemed non-significant by two-way ANOVA with Sidak’s Multiple comparison test.

Following antigen uptake, protein antigens are processed into peptides by lysosomal degradation [27]. This process involves a complex series of events resulting in fusion of the phagocytic endosome with the acid rich lysosome. Perturbations of the lysosomal degradation pathway are associated with a variety of inflammatory and degenerative diseases [28]. To assess antigen processing, BMDCs were incubated with ovalbumin-AF594 coated beads for 0–5 hours at 37°C. Internalised beads were identified by the absence of staining using rabbit anti-ovalbumin. To determine antigen degradation, loss of ovalbumin fluorescence was assessed by flow cytometry. Over time, the intensity of ovalbumin-AF594 fluorescence reduced (Fig 4A), indicative of antigen degradation [29]. However, the amount of degradation (Fig 4A) and the proportion of internalised beads with degraded ova (Fig 4B) were similar for WT and *Ptpn22*^*-/-*^ BMDC, indicating that Ptpn22 is dispensable for antigen processing.

We next assessed whether Ptpn22 was required for loading of peptides onto MHC class II. WT and *Ptpn22*^*-/-*^ BMDC were incubated for 18 hours at 37°C with a soluble fusion protein containing the Eα peptide epitope, GFP-Eα. Cell surface I-A^b^ restricted presentation of Eα peptide was determined by staining with anti-YAe-biotin (specific for the Eα_52–68_:IA^b^ complex), followed by streptavidin-APC [30]. Although Eα_52–68_:IA^b^ was detected on more than 50% of BMDC, the absence of Ptpn22 expression had no effect on the ability of BMDC to present Eα peptides (Fig 4C). In addition, cell surface expression of I-A^b^ was similar between WT and *Ptpn22*^*-/-*^ BMDC [31]. Having established that antigen degradation and loading onto MHC class II were similar between WT and *Ptpn22*^*-/-*^ BMDC, we next assessed if Ptpn22 altered the formation of DC:T-cell conjugates. BMDC were activated overnight with LPS to upregulate cell surface MHC class II expression, pulsed with OVA_323-339_ peptide, and labelled with CellTrace Far Red (CTFR). CTFR labelled BMDC were then co-cultured with CellTrace Violet (CTV) labelled WT OT-II T-cells for up to 120 mins at 37°C. The proportion of CTV^+^ CTFR^+^ cells increased over time, in keeping with the formation of DC:T-cell conjugates (Fig 4D). However, we did not observe any difference in the capability of WT and *Ptpn22*^*-/-*^ BMDC to form conjugates (Fig 4E), indicating that Ptpn22 is dispensable for DC:T-cell conjugate formation when *Ptpn22* deficiency is confined to the DC. Together these data indicate that Ptpn22 does not regulate antigen phagocytosis, processing and presentation.

### Ptpn22 is redundant for dendritic cell activation of antigen specific T-cells

**Fig 5:**
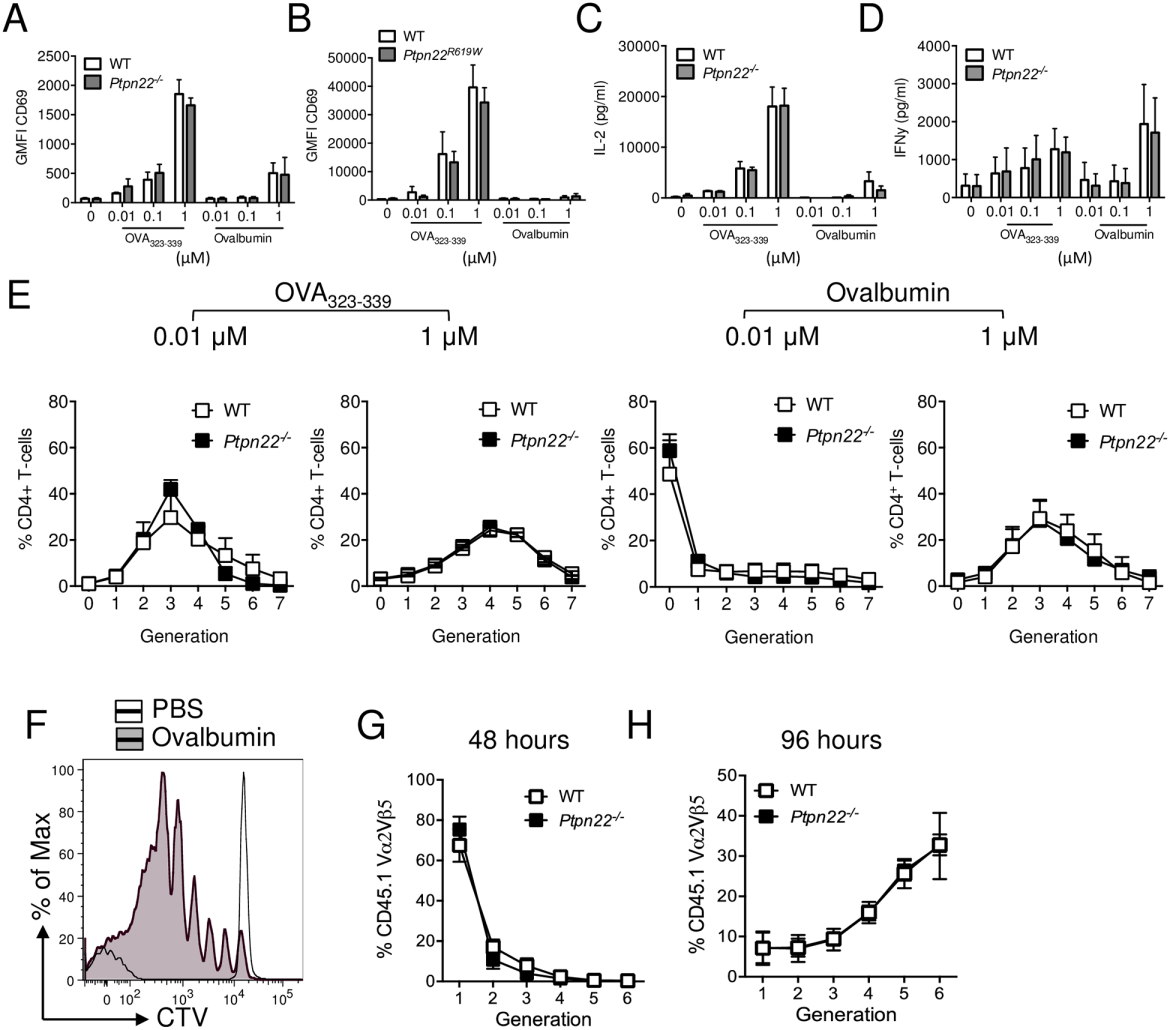
Ptpn22 variants do not modulate BMDC dependent OT.II T-cell activation. **(A-E)** Wild type (WT), *Ptpn22*^*-/-*^ and *Ptpn22*^*R619W*^ BMDC were stimulated overnight in the presence or absence of OVA_323-339_ (0.01–1 μM) or ovalbumin (0.01–1 μM). BMDC were harvested and co-cultured with CellTrace Violet (CTV) labelled CD4^+^ OT.II T-cells at a 1:2 BMDC:T-cell ratio. **(A-B)** 24 hour Geometric Mean Fluorescent Intensity (GMFI) surface expression of CD69 determined on live, singlet, CD4^+^ T-cells. **(A)** N = 3 independent experiments and **(B)** N = 4 independent experiments; bars represent mean ± s.d. **(C-D)** Co-culture supernatants were assessed for **(C)** IL-2 after 24 hours and **(D)** IFNγ after 144 hours. N = 3 independent experiments; bars represent mean ± s.d. **(E)** At day 6 the proportion of CD4^+^ T-cells within each CTV generation was determined by flow cytometry. N = 3 independent experiments, lines represent mean ± s.d. **(F-H)** CTV labelled CD45.1^+^ CD4^+^ TCR Vα2^+^Vβ5^+^ OT.II T-cells were adoptively transferred i.v. into CD45.2^+^ WT or *Ptpn22*^*-/-*^ recipient mice followed by i.p. immunisation of PBS or ovalbumin (100 μg/mouse). Spleens were assessed after 48h **(G)** or 96h **(F, H)** for CTV dilution within the CD45.1^+^ CD4^+^ TCR Vα2^+^Vβ5^+^ population by flow cytometry. **(F)** Representative flow cytometry plot showing CTV dilution following i.p. of PBS (black dashed line) or 10 μg ovalbumin (black line, grey fill). **(G-H)** Lines represent mean ± s.d., N = 3 **(G)** and N = 5 **(H)**. Differences between genotypes **(A-E, G, H)** were deemed non-significant by two-way ANOVA with Sidak’s Multiple comparison test.

It has previously been reported that *Ptpn22*^*R619W*^ BMDC are able to enhance antigen specific T-cell activation [9,12]. Given that we found no evidence that Ptpn22 regulated antigen phagocytosis, processing and presentation, we next sought to assess if APC induced T-cell activation is indeed dependent on Ptpn22. We compared the capability of WT and *Ptpn22*^*-/-*^ BMDC pulsed overnight with ovalbumin or OVA_323-339_ peptide at concentrations between 0.01–1 μM in the absence of TLR stimulation. BMDC were harvested, washed and co-cultured with WT CTV labelled CD4^+^ OT-II for up to 6 days. We observed that increasing concentrations of ovalbumin or OVA_323-339_ increased expression of T-cell early activation markers CD69 and CD25 (Fig 5A and panel A in S4 Fig), with OVA_323-339_ being more potent. WT and *Ptpn22*^*-/-*^ BMDC were equally capable of inducing the upregulation of both CD69 and CD25 regardless of peptide or protein concentration. Moreover, comparison of *Ptpn22*^*R619W*^ BMDC to WT controls did not reveal any differences in the capability to induce early T-cell activation (Fig 5B and panel B in S4 Fig). IL-2 is rapidly secreted by T-cells following T-cell activation. Although IL-2 secretion increased, depending on the dose of ovalbumin peptide or protein, no differences between BMDC genotypes were observed (Fig 5C and panel C in S4 Fig). In addition we observed no Ptpn22 dependent difference in IFNγ secretion over the 6 days of co-culture (Fig 5D). Surprisingly, and contrary to previous reports [9], no differences were observed in the capability of Ptpn22 variant DC to induce WT T-cell proliferation at multiple peptide or protein concentrations when compared to WT BMDC (Fig 5E and panels D and E in S4 Fig) or when BMDC were matured in the presence of LPS (Panel F in S4 Fig). This is in contrast to the enhanced T-cell proliferation observed when Ptpn22 variants are expressed in the T-cell compartment [17,32].

Finally, we sought to address if Ptpn22 was dispensable for APC mediated activation of T-cells *in vivo*. To address this, CTV labelled WT CD4^+^ CD45.1^+^ OT-II T-cells were adoptively transferred into CD45.2^+^ WT or *Ptpn22*^*-/-*^ mice and immunised with ovalbumin. No difference in *ex vivo* CD11c^+^ MHC class II IA^b+^ CD40 or CD86 expression was observed (Panels A and B in S5 Fig). 48 or 96 hours after immunisation, splenic CD45.1^+^ TCR Vα2^+^Vβ5^+^ CD4^+^ T-cells were assessed for CTV dilution by flow cytometry (Fig 5F). Consistent with our *in vitro* experiments, we observed no differences in the capability of *Ptpn22*^*-/-*^ APC to enhance the proliferation of adoptively transferred WT OT-II T-cells at either time point (Fig 5G and 5H). Furthermore no difference in OT-II T-cell proliferation was observed when T-cells were transferred into WT vs *Ptpn22*^*R619W*^ mice (Panel C in S5 Fig). Together these data demonstrate that Ptpn22 and the autoimmune associated *Ptpn22*^*R619W*^ variant does not impact the uptake, processing, and presentation of antigen by DCs, and that under non-inflammatory conditions the resultant T-cell priming is unaltered.

## Discussion

*PTPN22*^*R620W*^ is associated with an enhanced risk of developing multiple autoimmune diseases and, as such, has prompted investigations into which cells and pathways may be perturbed by carrying the polymorphism, thereby promoting autoimmunity. Adaptive immune responses are critical to the pathogenesis of autoimmune diseases associated with the *PTPN22*^*R620W*^ polymorphism. Furthermore, SFK dependent pathways regulate antigen processing and presentation in dendritic cells. Here we took a systematic approach to assess if Ptpn22 variants are capable of regulating APC antigen uptake, processing, presentation and APC-driven T-cell activation under non-inflammatory conditions. Unexpectedly, we observed that Ptpn22 and the autoimmune associated variant are dispensable for each of these processes both *in vitro* and *in vivo*.

Despite antigen uptake requiring a balance of SFK dependent phosphorylation and phosphatase-regulated dephosphorylation, our data indicate that Ptpn22 is dispensable in this process. In contrast to a previous report observing enhanced uptake of *E*.*coli* by *Ptpn22*^*R619W*^ macrophages [14], we observed no differences in receptor-mediated antigen uptake by dendritic cells across a range of receptor pathways. The cause of this discrepancy may reflect differences in cell-mediated uptake in macrophages versus dendritic cells, or may reflect differences in mouse health status/microbiota, or may indicate that Ptpn22 specifically regulates *E*.*coli* dependent uptake rather than broadly regulating receptor-mediated endocytosis pathways. Overall, our combined data strongly support the conclusion that alternative phosphatases are likely to operate as primary, or redundant regulators of antigen processing pathways. Indeed, phosphatases including SHIP-1, SHP-1, and CD45 have all been observed to regulate processes required for antigen uptake and processing [15,33,34].

Again contrary to previous reports [9,12], we observed no difference in the capability of Ptpn22 deficient or *Ptpn22*^*R619W*^ BMDC or splenic DC versus the equivalent WT-derived populations to induce WT OT-II T-cell proliferation. The reason for this observed discrepancy in T-cell activation compared with previous studies is unclear. It may be due to subtle differences in the thresholds for signalling dependent on the dosing concentrations of BMDC stimuli, the age of animals studied or the possibility that previous work utilised cells from animals with active inflammatory changes. Most strikingly, and consistent with the interpretation that Ptpn22 has little impact on APC function in a physiological setting, our *in vivo* data demonstrated equivalent activation of WT OT-II CD4^+^ T-cells following adoptive transfer into Ptpn22 deficient or WT control mice (Fig 5G and 5H). As we, and others, have previously reported, *Ptpn22*^*R619W*^ mice exhibit increased responses to ova immunisation and marked changes in T-cell activation [4,17,35]. Thus, although our data revealed no difference in conjugate formation between WT and Ptpn22 variant DC with WT T-cells, Ptpn22 is likely to modulate MHC class II restricted DC:T-cell conjugate formation in a T-cell dependent manner (rather than through effects on the DC). Consistent with this idea, Ptpn22 deficient CD8^+^ OT-I T-cells have an enhanced capability to form conjugates with WT APCs in response to low affinity peptide antigen, leading to enhanced T-cell activation in an LFA-1 dependent manner [5,32].

In conclusion we show that Ptpn22 deficiency or the expression of the autoimmune associated variant of Ptpn22 does not regulate the phagocytic capability of *in vitro* generated BMDC or splenic DCs. Furthermore, as systematically demonstrated here, using a broad range of models, Ptpn22 is redundant for the processing and presentation of protein antigen and that Ptpn22 variant DC were not altered in their capability to induce T-cell activation under non-inflammatory conditions either *in vitro* or *in vivo*. Antigen uptake, processing and presentation are fundamental to the induction of adaptive T-cell responses. Our data reveal that perturbations caused by the autoimmune associated risk variant of Ptpn22 are unlikely to promote autoimmunity via these pathways in DC.

## Supporting information

S1 Fig WT, *Ptpn22*^*-/-*^ *and Ptpn22*^*R619W*^ genotyping and expression. **(A)** Day 6 WT, *Ptpn22*^*-/-*^ and *Ptpn22*^*R619W*^ BMDC were lysed and DNA extracted. Ptpn22 PCR product was run on a 1% agarose gel and WT and *Ptpn22*^-/-^ bands were identified (498bp and 221bp respectively). **(B)** RNA was extracted and cDNA synthesised from day 6 WT, *Ptpn22*^*-/-*^ *and Ptpn22*^*R619W*^ BMDC. Expression of Ptpn22 was determined by real-time PCR and normalised to expression of 18S. Bars represent the mean of 3 individual mice + s.d.
(PDF)

S2 Fig WT and *Ptpn22*^*-/-*^ generate phenotypically similar immature BMDC. Day 6 WT and *Ptpn22*^*-/-*^ BMDC were harvested and cell surface stained for CD11c, CD11b, and MHC class II I-A^b^ and the proportion of MHC class II^+^ CD11c^+^, MHC class II^Hi^ CD11b^Int^ DC, and MHC class II^Int^ CD11b^HI^ macrophages determined by flow cytometry. **(A)** Representative gating of live, singlets, followed by CD11c^+^ MHC class II^+^ gate (left plot), followed by DC gate (CD11b^Int^ MHC class II^HI^) and macrophage gate (CD11b^HI^ MHC class II^Int^). **(B)** Data show percentages of each population within WT and *Ptpn22*^*-/-*^ BMDC cultures. Data are of 8 independent experiments. Bars represent mean + s.d. Differences between genotypes were deemed non-significant by two-way ANOVA with Sidak’s Multiple comparison test.
(PDF)

S3 Fig Receptor mediated endocytosis is similar between WT and *Ptpn22*^*-/-*^ BMDC. **(A)** Day 6 WT and *Ptpn22*^*R619W*^ BMDC were harvested and cell surface stained for CD206. Live singlet CD11c^+^ cells were gated and CD206 Geometric Mean Fluorescent Intensity (GMFI) determined by flow cytometry. N = 3 independent experiments; bars represent mean + s.d. **(B)** WT and *Ptpn22*^*-/-*^ BMDC were incubated with labelled heat killed *L*. *monocytogenes* (HKLM) at 37°C for 0–60 minutes. The percentage of CD11c^+^ HKLM^+^ BMDC was determined by flow cytometry. N = 5 independent experiments; bars represent mean + s.d. **(C)** Day 6 BMDC were generated from WT or *Ptpn22*^*-/-*^ mice. BMDC were incubated with labelled heat killed *C*. *albicans* (HKCA) at 4°C or 37°C for 1 hour. The percentage of CD11c^+^ HKCA^+^ BMDC was determined by flow cytometry. N = 4; bars represent mean + s.d. Differences between genotypes were deemed non-significant by unpaired T-test **(A, C)** and two-way ANOVA with Sidak’s Multiple comparison test **(B)**.
(PDF)

S4 Fig *Ptpn22*^*R619W*^ does not alter BMDC induced T-cell activation. WT, *Ptpn22*^*-/-*^ and *Ptpn22*^*R619W*^ BMDC were stimulated overnight in the presence or absence of OVA_323-339_ (0.01–1 μM) or ovalbumin (0.01–1 μM). BMDC were harvested and co-cultured with CellTrace Violet (CTV) labelled CD4^+^ OT-II T-cells at a 1:2 BMDC:T-cell ratio. **(A-B)** 24 hour Geometric Mean Fluorescent Intensity (GMFI) surface expression of CD25 determined on live, singlet, CD4^+^ T-cells. **(A)** N = 3 independent experiments; **(B)** N = 4 independent experiments; bars represent mean ± s.d. **(C)** Co-culture supernatants were assessed for IL-2 after 24 hours. N = 4 independent experiments; bars represent mean + s.d. **(D-E)** WT and *Ptpn22*^*R619W*^ BMDC pulsed overnight with **(D)** OVA_323-339_ (1 μM) or **(E)** ovalbumin (1 μM) were co-cultured with CTV labelled CD4^+^ OT-II T cells. At day 6 the proportion of CD4^+^ T-cells within each CTV generation was determined by flow cytometry. N = 4 independent experiments; lines represent mean ± s.d. Differences between genotypes were deemed non-significant by two-way ANOVA with Sidak’s Multiple comparison test. **(F)** WT and *Ptpn22*^*-/-*^ BMDC were stimulated overnight in the presence or absence LPS in the presence of ovalbumin (1μM). BMDC were harvested and co-cultured with CTV labelled CD4^+^ OT-II T-cells at a 1:2 BMDC:T-cell ratio. At day 6 the proportion of CD4^+^ T-cells within each CTV generation was determined by flow cytometry N = 7 independent experiments; bars represent mean + s.d.
(PDF)

S5 Fig Ptpn22 variants do not modulate BMDC dependent OT-II T-cell activation. **(A-B)** Splenocytes from WT or *Ptpn22*^*-/-*^ mice were surface stained and mean fluorescent intensity of CD40 and CD86 on live, singlet, Lin^-^, CD11c^+^, MHC class II IA^b+^ cells was determined by flow cytometry. Bars represent mean ± s.d, each point represents an individual mouse. **(C)** CTV labelled CD45.1^+^ CD4^+^ TCR Vα2^+^Vβ5^+^ OT.II T-cells were adoptively transferred i.v. into CD45.2^+^ WT or *Ptpn22*^*R619W*^ recipient mice followed by i.p. immunisation of PBS or ovalbumin (100 μg/mouse). Spleens were assessed after 96h for CTV dilution within the CD45.1^+^ CD4^+^ TCR Vα2^+^Vβ5^+^ population by flow cytometry. Bars represent mean + s.d., N = 2/3 per group.
(PDF)
