# Supplementary figures and images for: Protein tyrosine phosphatase PTPN22 is dispensable for dendritic cell antigen processing and promotion of T-cell activation by dendritic cells
Source: PLoS One. 2017 Oct 17;12(10):e0186625. doi: 10.1371/journal.pone.0186625 (PMC5645108; doi:10.1371/journal.pone.0186625)

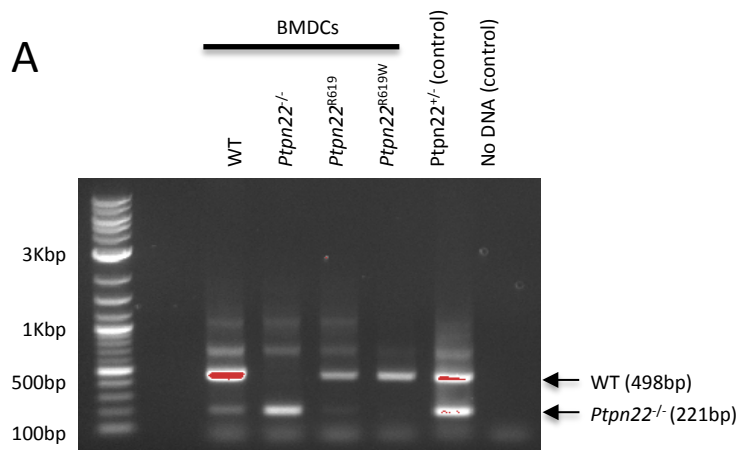

**B**

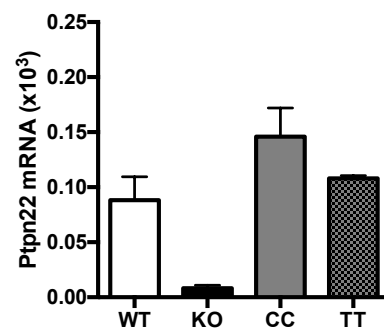

Supplement: S1 Fig — (A) Day 6 WT, Ptpn22-/- and Ptpn22R619W BMDC were lysed and DNA extracted. Ptpn22 PCR product was run on a 1% agarose gel and WT and Ptpn22-/- bands were identified (498bp and 221bp respectively). (B) RNA was extracted and cDNA synthesised from day 6 WT, Ptpn22-/- and Ptpn22R619W BMDC. Expression of Ptpn22 was determined by real-time PCR and normalised to expression of 18S. Bars represent the mean of 3 individual mice + s.d. (PDF) [file pone.0186625.s001.pdf]

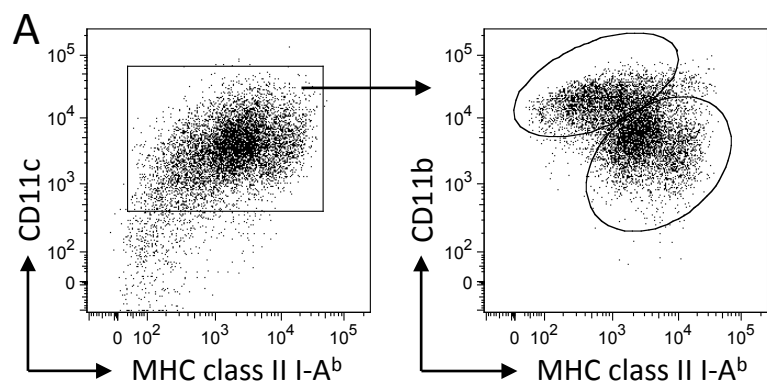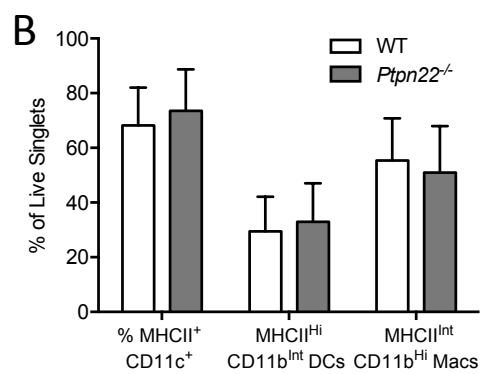

Supplement: S2 Fig — Day 6 WT and Ptpn22-/- BMDC were harvested and cell surface stained for CD11c, CD11b, and MHC class II I-Ab and the proportion of MHC class II+ CD11c+, MHC class IIHi CD11bInt DC, and MHC class IIInt CD11bHI macrophages determined by flow cytometry. (A) Representative gating of live, singlets, followed by CD11c+ MHC class II+ gate (left plot), followed by DC gate (CD11bInt MHC class IIHI) and macrophage gate (CD11bHI MHC class IIInt). (B) Data show percentages of each population within WT and Ptpn22-/- BMDC cultures. Data are of 8 independent experiments. Bars represent mean + s.d. Differences between genotypes were deemed non-significant by two-way ANOVA with Sidak’s Multiple comparison test. (PDF) [file pone.0186625.s002.pdf]

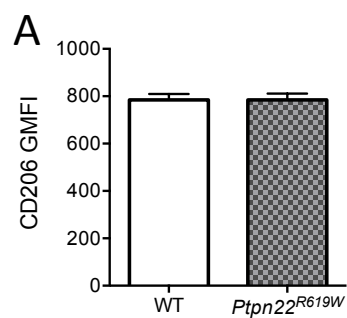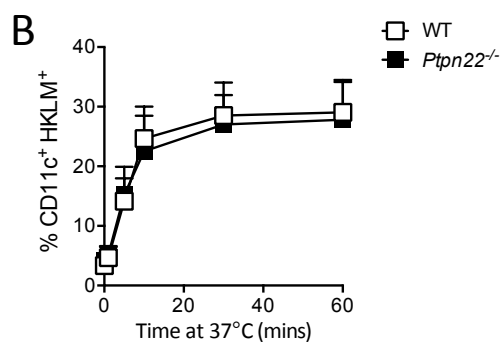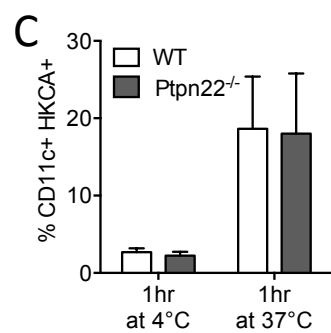

Supplement: S3 Fig — (A) Day 6 WT and Ptpn22R619W BMDC were harvested and cell surface stained for CD206. Live singlet CD11c+ cells were gated and CD206 Geometric Mean Fluorescent Intensity (GMFI) determined by flow cytometry. N = 3 independent experiments; bars represent mean + s.d. (B) WT and Ptpn22-/- BMDC were incubated with labelled heat killed L. monocytogenes (HKLM) at 37°C for 0–60 minutes. The percentage of CD11c+ HKLM+ BMDC was determined by flow cytometry. N = 5 independent experiments; bars represent mean + s.d. (C) Day 6 BMDC were generated from WT or Ptpn22-/- mice. BMDC were incubated with labelled heat killed C. albicans (HKCA) at 4°C or 37°C for 1 hour. The percentage of CD11c+ HKCA+ BMDC was determined by flow cytometry. N = 4; bars represent mean + s.d. Differences between genotypes were deemed non-significant by unpaired T-test (A, C) and two-way ANOVA with Sidak’s Multiple comparison test (B). (PDF) [file pone.0186625.s003.pdf]

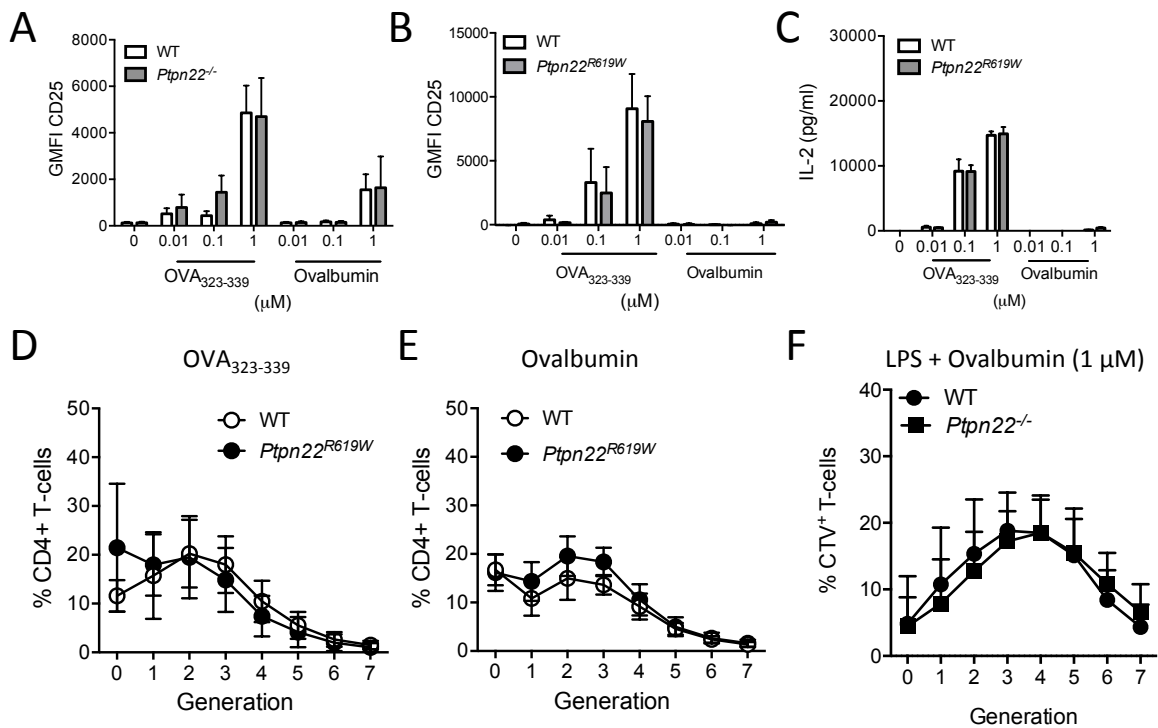

Supplement: S4 Fig — WT, Ptpn22-/- and Ptpn22R619W BMDC were stimulated overnight in the presence or absence of OVA323-339 (0.01–1 μM) or ovalbumin (0.01–1 μM). BMDC were harvested and co-cultured with CellTrace Violet (CTV) labelled CD4+ OT-II T-cells at a 1:2 BMDC:T-cell ratio. (A-B) 24 hour Geometric Mean Fluorescent Intensity (GMFI) surface expression of CD25 determined on live, singlet, CD4+ T-cells. (A) N = 3 independent experiments; (B) N = 4 independent experiments; bars represent mean ± s.d. (C) Co-culture supernatants were assessed for IL-2 after 24 hours. N = 4 independent experiments; bars represent mean + s.d. (D-E) WT and Ptpn22R619W BMDC pulsed overnight with (D) OVA323-339 (1 μM) or (E) ovalbumin (1 μM) were co-cultured with CTV labelled CD4+ OT-II T cells. At day 6 the proportion of CD4+ T-cells within each CTV generation was determined by flow cytometry. N = 4 independent experiments; lines represent mean ± s.d. Differences between genotypes were deemed non-significant by two-way ANOVA with Sidak’s Multiple comparison test. (F) WT and Ptpn22-/- BMDC were stimulated overnight in the presence or absence LPS in the presence of ovalbumin (1μM). BMDC were harvested and co-cultured with CTV labelled CD4+ OT-II T-cells at a 1:2 BMDC:T-cell ratio. At day 6 the proportion of CD4+ T-cells within each CTV generation was determined by flow cytometry N = 7 independent experiments; bars represent mean + s.d. (PDF) [file pone.0186625.s004.pdf]

**A**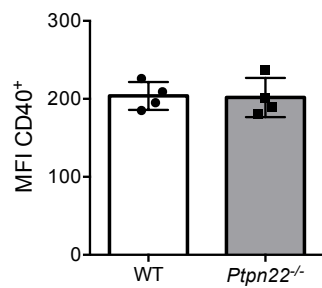**B**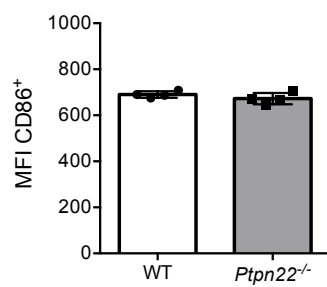**C**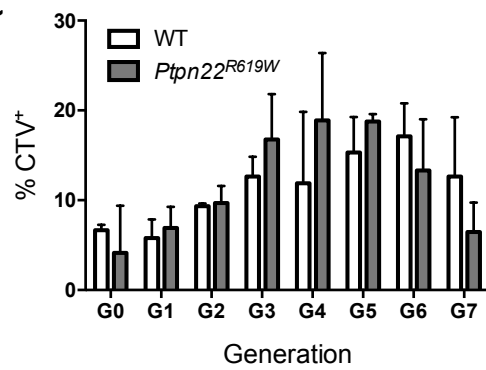

Supplement: S5 Fig — (A-B) Splenocytes from WT or Ptpn22-/- mice were surface stained and mean fluorescent intensity of CD40 and CD86 on live, singlet, Lin-, CD11c+, MHC class II IAb+ cells was determined by flow cytometry. Bars represent mean ± s.d, each point represents an individual mouse. (C) CTV labelled CD45.1+ CD4+ TCR Vα2+Vβ5+ OT.II T-cells were adoptively transferred i.v. into CD45.2+ WT or Ptpn22R619W recipient mice followed by i.p. immunisation of PBS or ovalbumin (100 μg/mouse). Spleens were assessed after 96h for CTV dilution within the CD45.1+ CD4+ TCR Vα2+Vβ5+ population by flow cytometry. Bars represent mean + s.d., N = 2/3 per group. (PDF) [file pone.0186625.s005.pdf]
